# Peritonitis Caused by Various Species of Diaporthe in Peritoneal Dialysis Patients: A Plant Pathogen to Human Infection

**DOI:** 10.7759/cureus.57016

**Published:** 2024-03-27

**Authors:** Guttiga Halue, Rutchanee Chieochanthanakij, Thanapat Kittipanyaworakun, Panthira Passorn, Donkum Kaewboonsert, Tanyalak Tharavichitkul, Athiphat Banjongjit, Talerngsak Kanjanabuch, Somchai Eiam-Ong

**Affiliations:** 1 Department of Medicine, Phayao Hospital, Phayao, THA; 2 Dialysis Unit, Sawanpracharak Hospital, Nakhon Sawan, THA; 3 Division of Nephrology, Department of Medicine, Chiangrai Prachanukroh Hospital, Chiangrai, THA; 4 Nephrology Unit, Vichaiyut Hospital, Bangkok, THA; 5 Division of Nephrology, Department of Medicine, Faculty of Medicine, Chulalongkorn University, Bangkok, THA; 6 Center of Excellence in Kidney Metabolic Disorders, Faculty of Medicine, Chulalongkorn University, Bangkok, THA; 7 Continuous Ambulatory Peritoneal Dialysis (CAPD) Excellent Center, King Chulalongkorn Memorial Hospital, Chulalongkorn University, Bangkok, THA

**Keywords:** diaporthe phaseolorum, peritonitis, peritoneal dialysis, diaporthe eucalyptorum, diaporthe amygdali

## Abstract

Peritonitis caused by dematiaceous molds is uncommon but poses a significant threat to patients undergoing peritoneal dialysis (PD), leading to high mortality and morbidity. This report highlights three cases of peritonitis caused by three distinct species of* Diaporthe *(*D. amygdali, D. eucalyptorum, *and* D. phaseolorum*), initially unidentified through conventional culture methods. The nucleotide sequences of internal transcribed spacer regions (ITS), 18S nuclear ribosomal small subunit (SSU), and 28S nuclear ribosomal large subunit (LSU) of the ribosomal DNA gene correctly identified the isolates. Despite early catheter removal and administration of appropriate antifungal medications, all patients experienced fatal outcomes. DNA barcoding emerges as a valuable tool for accurately diagnosing species within the genus of pathogenic microbes, aiding in identifying the root causes of infections. It emphasizes the importance of strict adherence to aseptic techniques during PD exchanges to prevent peritonitis caused by plant-borne pathogens.

## Introduction

Fungal peritonitis, though relatively rare compared to bacterial peritonitis, carries higher mortality and worse outcomes [1,2]. While *Candida* spp. is the most common fungal species implicated in peritoneal dialysis (PD)-related fungal peritonitis, a growing trend of mold infections has been noted, particularly in tropical countries [2]. Among mold, PD-related peritonitis, explicitly caused by dematiaceous mold, is not a well-documented infection in the medical literature. The term "dematiaceous mold" refers to molds with a dark appearance due to pigmented spores or mycelia. Although several dematiaceous molds have been reported to cause PD-related peritonitis, only one case has been attributed to *Diaporthe*, with limited clinical details and species identification. Therefore, this report presents three cases of PD-associated peritonitis caused by genetically confirmed *Diaporthe* spp., emphasizing the importance of genetic confirmation in clinical practice.

## Case presentation

This section details three cases of PD-related peritonitis caused by *Diaporthe* spp. All isolations were identified through DNA barcoding, revealing different *Diaporthe* species. Traditional culture methods were unable to identify the species but provided information on antifungal susceptibility patterns, indicating resistance to all available antifungal agents except amphotericin B (AMB) and voriconazole (VCZ). Minimum inhibitory concentrations (MIC) values for various antifungal agents were determined using the modified Clinical and Laboratory Standards Institute (CLSI) M38-A broth microdilution method [3] with reference to *Aspergillus* spp [4]. Table 1 summarizes the results of DNA barcoding and susceptibility tests.

**Table 1:** Results of DNA barcoding and susceptibility tests Species were confirmed by DNA barcoding using nucleotide sequences of internal transcribed spacer (ITS: ITS1/ITS4 primer [5]) small subunit region (SSU: NS1/NS2 primer [5]) and large subunit region (LSU: NL-1/NL-4 primer [6]) of the ribosomal DNA gene. The purified PCR products were then outsourced for Sanger sequencing service (First BASE Laboratories, Singapore Science Park II, Singapore). The sequencing results were subjected to BLASTN (National Center for Biotechnology Information Internet homepage) search against the GenBank database for homology identities. Antifungal susceptibility patterns of the mold against common antifungal medications were assessed by the broth dilution technique (according to the Clinical and Laboratory Standards Institute (CLSI) document M38-A2 protocol) with reference to *Aspergillus spp*. AMB, amphotericin B; CAS, caspofungin; FLC, fluconazole; ISZ, isavuconazole; ITZ, itaconazole; PSZ, Posaconazole; TBF=terbinafine; VCZ, voriconazole; (R), resistance; (S), susceptible; (N/A), non-available

No.	Hospital	Reported taxonomical identification (Gene bank accession number/Percent identity)			Age/gender	Underlying diseases	Activity/plant linked with the infection	Antifungal susceptibility results	Treatment	Outcomes
		ITS gene	LSU gene	SSU gene						
1.	Sawan Pracharak	*D. eucalyptorum *(MK396569.1/ 98.83%)	*D. eucalyptorum *(MK182372.1/ 99.78%)	*D. eucalyptorum* (MK981536.1/ 100%)	56/male	HT/DM	Agricultural worker	AMB =1 (S), VCZ =1 (S), FLC >64 (R), ITZ =4 (R), CAS =16 (R), ISZ =4 (R), TBF =4 (N/A), PSZ =8 (R)	AMB 50 mg- 25 days (until dead)	Death (D20)
2.	Phayao	-	*D. phaseolorum *(MH877680.1/ 100%)	*D. phaseolorum* (ON926922.1/100)	65/female	HT/DM	Agricultural worker	AMB =1 (S), VCZ =1 (S), FLC ≥64 (R), ITZ =1 (S), CAS >16 (R)	AMB 65 mg- 13 days (until dead)	Death (D27)
3.	Chiangrai Prachanukroh	*D. amygdali* (MT199853.1/ 94.14%)	Unidentified	*D. amygdali* (MH051054.1/ 87.47%)	60/male	HT	Caregiver, farmer	AMB =1 (S), VCZ =2 (S), FLC>64 (R), ITZ =4 (R), CAS =16 (R), ISC =4 (R), TBF =8 (N/A), PSZ =4 (R)	Palliative care	Death (D32)

Case 1

**Figure 1:**
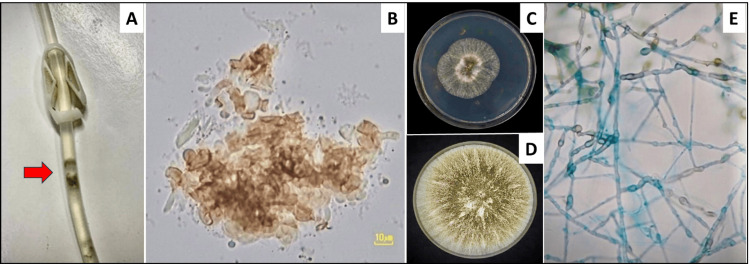
The black particles observed inside the PD catheter of patient Case #1 (A). A wet smear with potassium hydroxide (KOH) depicted brown mycelium (B). Diaporthe colony on SDA after 3 days (C) and 7 days (D) of incubation at 25°C. The colony was off-white in color, had a velvety texture, and darkened over time. (E) The conidia of Diaporthe with blue color by Lactophenol cotton blue staining with 1000x magnification.

A 56-year-old agricultural worker with hypertension and diabetic kidney failure on continuous ambulatory PD (CAPD 2-liter 1.5% dextrose bag for four exchanges daily for 15 months) presented with low-grade fever and abdominal pain on day 0. Despite initial treatment for bacterial peritonitis (initial peritoneal dialysis effluent (PDE) leukocyte count of 1,306 cells/µL of which 98% were neutrophils), refractory culture-negative peritonitis was diagnosed on day 12. Fungal peritonitis was suspected on day 12 based on a positive PDE galactomannan index (GMI 4.6, the cut-off level <0.5). Intravenous AMB deoxycholate at 1 mg/kg/day was initiated on the same day, and the PD catheter was removed on day 14. The patient transitioned to hemodialysis but deceased on day 20. Subsequent fungal culture grew an unidentified dematiaceous mold (Figure 1).

Case 2

A 65-year-old female agricultural worker (corn and soybean) with diabetic kidney failure on CAPD using a 2-liter 1.5% dextrose bag for four exchanges daily for 10 months presented with fatigue and cloudy PDE on day 0. She had no history of peritonitis and exit-site infection. Peritonitis was diagnosed with dialysate leukocyte counts of 815 cells/μL and neutrophils at 56%. She received empirical antibiotics with intraperitoneal cefazolin and ceftazidime (1 gm each daily) and fungal prophylaxis with fluconazole (200 mg/day). A positive GMI on day 5 PDE (4.8) was disclosed on day 8. Gray particles were observed inside the PD catheter, leading to the removal of the PD catheter on day 9, transferring to hemodialysis on day 10, and ultimately passing away on day 27. Fungal culture on day 23 identified an unidentified mold. The empirical antibiotics were replaced with intravenous deoxycholate AMB, 1 mg/kg/day on day 13, and continued until the patient’s death (day 13 to day 27).

Case 3

A 60-year-old unemployed male with hypertensive kidney failure on assisted CAPD using a 2-liter 1.5% dextrose bag for four exchanges daily for 7.5 years with a spouse caregiver who worked as a farmer. He presented with acute abdominal pain and cloudy effluent on day 0. The diagnosis of peritonitis was confirmed with a PDE leukocyte count of 478 cells/µl and neutrophils at 96%. Empirical antibiotics, including intraperitoneal cefazolin and ceftazidime at 1 gram daily each, were initiated. Ceftazidime-susceptible *A. baumannii* was isolated from the specimens on days 0 and 5. Ceftazidime was continued for four weeks alongside concurrent fluconazole prophylaxis. Despite initial treatment for bacterial peritonitis, refractory culture-negative peritonitis persisted. The PDE cell count remained above 100 cells/µl. On day 30, dark particles in the PD catheter and a positive GMI (0.85) suggested fungal peritonitis. The PD catheter was removed, and the patient transitioned to hemodialysis, succumbing on day 32. Fungal culture grew an unidentified dematiaceous mold.

## Discussion

This section presents three case reports of PD-related peritonitis caused by various strains of *Diaporthe*. The conventional culture method failed to identify the pathogens, highlighting the limitations of morphological identification in clinical microbiology labs and the resulting delayed treatment and fatal outcomes. Identifying rarely found molds based on morphology is challenging, prompting the use of DNA barcoding in the last decade for accurate fungal species identification, especially for discovering novel fungi from clinical specimens [7].

*Diaporthe* belongs to the Diaporthaceae family in the Division of Ascomycota [8]. While primarily affecting plants and associated with various plant diseases (e.g., grapes, soybean, sunflower, citrus, peach, tea mango, dragon fruit, rambutan, coffee bean, blue trumpet vine, red silk cotton, and makha) [9-12], *Diaporthe* species are rarely linked to human infections. These infections are typically opportunistic, occurring in individuals with compromised immune systems, such as those with organ transplants, HIV/AIDS, poorly controlled diabetes, or other immunosuppressive conditions [13].

These infections often manifest as localized skin or soft tissue infections, such as mycetoma and soft tissue ulcers, which are usually curable [13]. There has been only one reported case of PD-related peritonitis due to *Diaporthe*. In this instance, a Canadian female presented with PD-related peritonitis and fungal colonization in her PD catheter lumen after returning from Panama. Besides diabetes, she had no other compromised immune diseases. Unfortunately, the report did not provide details on the clinical course, laboratories, treatment, outcome, or specific nomenclature of the *Diaporthe* pathogen [14].

All three presented cases mimicked symptoms of bacterial peritonitis but showed negative bacterial cultures, leading to a diagnosis of refractory culture-negative peritonitis. Clues suggesting fungal infection included intraluminal colonization and elevated effluent GMI. Notably, patients/caregivers with agricultural backgrounds and activities in the Northern region of Thailand, where the pathogen is prevalent [10-12], were exposed to this fungus during work. They reported strict compliance with aseptic handwashing and peritoneal dialysis (PD) exchange instructions. Therefore, touch contamination during agricultural work is considered a potential root cause of these infections. It's noteworthy that our cases involved immunocompetent individuals, differing from the previously mentioned reports.

Current guidelines lack specific recommendations for *Diaporthe* infections, with general suggestions for antifungal treatment following catheter removal [1,15]. However, the dismal outcomes in the presented cases, despite susceptibility reports favoring amphotericin B, underscore the need for more targeted therapeutic strategies. The cases emphasize the importance of early detection and pathogen identification through molecular techniques and serology, guiding the root cause of infection, and determining treatment protocols.

Although all cases received prompt PD catheter removal per guidelines for fungal peritonitis [1], there was a delay from peritonitis onset to diagnosis, underscoring the importance of early detection. Despite several risk factors contributing to poor outcomes, including old age, diabetes, and high dialysate cell count [1,2], the pathogen's virulence might play a role. The cases highlight the urgency of early detection and identification through molecular techniques and serology, guiding treatment decisions and potentially saving lives [16-18].

Voriconazole emerges as a potential alternative for eradicating invasive *Diaporthe* infections due to its reasonable peritoneal fluid penetration and minimal peritoneal clearance [17,18]. Combining voriconazole with early PD catheter removal could be recommended for treating PD-related peritonitis caused by *Diaporthe*.

The 2021 Global Guideline for the diagnosis and management of rare mold infections, an initiative led by the European Confederation of Medical Mycology in collaboration with the International Society for Human and Animal Mycology and the American Society for Microbiology, provides antifungal regimens tailored to the species of fungi involved. Notably, intrinsic resistance to antifungal agents varies significantly among molds [15]. Genera such as *Fusarium*, *Lomentospora*, and *Scedosporium* are commonly multi-resistant to most currently available antifungals [15]. Unfortunately, no Genera *Diaporthe* is mentioned. Antifungal susceptibility testing for rare molds lacks standardization, and breakpoints are unavailable. Additionally, diagnosis through conventional microscopy and culture poses challenges due to subjective interpretation. Consequently, accurate identification of rare pathogens using DNA barcoding can guide clinical decision-making and potentially improve patient outcomes.

## Conclusions

These three case reports underscore the importance of molecular identification and serologic methods for the early detection and identification of *Diaporthe* in PD-related peritonitis. Strict adherence to aseptic techniques during PD exchanges is crucial to prevent the risk of plant-borne fungal infections. Further research is warranted to establish specific treatment protocols for *Diaporthe* infections in PD patients.
